# Deficits in Motion and Form Perception in Infantile Nystagmus Syndrome

**DOI:** 10.1167/iovs.66.13.44

**Published:** 2025-10-27

**Authors:** Mahesh R. Joshi, Aastha Subedi, Gyan B. Basnet, Asma A. A. Zahidi, Hari B. Adhikari

**Affiliations:** 1 School of Health Professions, University of Plymouth, Plymouth, United Kingdom; 2 Himalaya Eye Institute, Pokhara University, Pokhara, Nepal

**Keywords:** nystagmus, motion perception, shape perception

## Abstract

**Purpose:**

Visual deficits in infantile nystagmus syndrome (INS) could be a result of retinal blur from excessive eye movements and/or cortical changes from visual deprivation. We measured global motion and form sensitivity in INS to compare deficits between motion and form perception and to decipher the role of internal noise (local deficit such as eye movement) and sampling efficiency (global cortical deficit).

**Methods:**

A total of 30 participants (14.40 ± 4.83 years) with INS and 30 age-matched controls discriminated the direction of motion and orientation of physically equivalent translational random dot kinematograms (RDKs) and Glass patterns. Both stimuli consisted of 240 black dots (RDKs) and 120 pairs of dipoles (Glass patterns) with a display duration of 1.0 second. Two experimental paradigms were employed: coherence threshold (random noise) and equivalent noise (at five external noise levels).

**Results:**

The mean motion coherence thresholds at 5°/s and 10°/s were higher in INS (50.55% ± 21.33% and 31.87% ± 14.69% respectively) compared to controls (24.04% ± 13.22% and 20.65% ± 12.89, respectively) (*P* < 0.01). The mean orientation coherence thresholds were also higher in INS (12.23% ± 0.32% vs. 7.88% ± 0.33%; *P* < 0.01). For the equivalent noise paradigm, thresholds were higher for INS at no noise and 3 lower noise levels (*P* < 0.01), but similar at the highest noise level (*P* > 0.01). Higher internal noise best explained the difference in performance between INS and controls for both motion and form (*P* > 0.05).

**Conclusions:**

INS results in lower sensitivity to both motion and form perception. These deficits are due to higher internal noise, which could arise from early areas of visual processing such as primary visual cortex as a result of abnormal eye movement or effect of early visual deprivation.

Infantile nystagmus syndrome (INS) includes various congenital conditions characterized by the onset of abnormal eye movements in early infancy (0–6 months).^1^^,^^2^ INS can occur in the absence of any pathology (idiopathic nystagmus) or in association with conditions such as albinism and congenital cataract. Irrespective of the underlying cause, INS results in abnormal eye movement characterized by horizontal pendular/jerk eye movements.^2^

INS is associated with reduced sensitivity to a wide spectrum of visual functions, such as visual acuity (VA),^3^ contrast sensitivity, motion perception,^4^^–^^6^ and crowding effects.^7^^–^^9^ Two main factors have been discussed as the cause of these visual defects: (1) fixational instability and smearing of retinal images due to abnormal eye movements, and (2) effect of visual deprivation due to blurry retinal images in early phases of visual development.^2^^,^^10^^,^^11^ The smearing and fixation instability should impact the stimuli presented along the horizontal meridian more than the vertical as the direction of abnormal eye movement is mostly horizontal in INS. Poor performance along horizontal meridians has been reported for crowding,^7^ orientation discrimination,^12^ and moving sinusoidal gratings.^4^

Multiple temporally different images on retina combined with abnormal eye movement when observing moving objects could cause greater fixational instability and image smearing compared to static stimuli in nystagmus. Only few studies have systematically investigated motion perception in nystagmus using random dot kinematograms (RDKs),^5^ Gabor patches,^6^ and sinusoidal gratings.^4^ These studies report elevated motion thresholds in nystagmus. In terms of difference in performance along the horizontal and vertical meridians, a study using sinusoidal gratings showed a difference between the two meridians,^4^ but other studies using RDK^5^ and Gabor patches^6^ reported no difference in the scale of deficits between the meridians. The motion deficits in nystagmus are also observed at the null point where eye movements are dampened.^5^ Similarly, visual acuity deficits also persist when the image blur on retina is minimized using very brief stimuli (<1 ms).^12^ Taken together, these findings suggest that abnormal eye movement cannot fully explain the range and magnitude of deficits observed in nystagmus.^5^^,^^10^

Visual deprivation in infancy due to retinal image smearing and fixational instability that disrupts normal visual development could be the main cause of visual deficits in INS.^10^^,^^11^ The constant retinal motion at or near birth hinders clear retinal image formation during the critical developmental period, resulting in bilateral deprivation amblyopia.^8^^,^^13^ The effect of early deprivation could explain the spectrum of deficits in nystagmus based on similar mechanisms as those observed in amblyopia. In addition, it has been suggested that the abnormal eye movements may not be the cause of visual deficits in INS but instead an adaptive mechanism to already reduced visual sensitivity due to early deprivation.^11^ The pendular oscillation of eyes in INS could be an adaptive mechanism to maintain an image near the fovea to improve visual functioning.^11^ One method to decipher the role of abnormal eye movements on motion perception psychophysically is to compare performance for moving stimuli with static form stimuli that are equivalent in all aspects except for the presence and absence of motion. If object motion causes larger deterioration in performance due to its interaction with abnormal eye movements, then we would expect larger deficits for motion compared to form (orientation) tasks. Alternatively, if the origin of deficits is mainly in higher cortical areas, as previously reported for different amblyopia types,^14^^,^^15^ they could affect both motion and shape perception. In this study, we measured performance in nystagmus along motion and form domains using physically equivalent RDKs and Glass patterns.

Motion and form processing occurs in two stages. The local direction and orientation of individual elements that create a visual scene are first processed in the early areas of the visual system, such as the primary visual cortex (V1). This is then followed by the integration of local cues to provide a global percept of the whole scene at higher cortical areas such as the middle temporal (MT) and medial superior temporal (MST) for motion and the V4 for form.^16^^–^^18^ Such a notion of hierarchical processing is supported by the neuronal properties at these cortical areas where direction- and orientation-selective neurons in the V1 with their smaller receptive fields are well tuned to process local properties, whereas the neurons in the MT, MST, and V4, with their larger receptive fields, are required for global processing.^18^^–^^21^ Although studies have reported motion processing deficits in nystagmus, only one study has attempted to assess the role of local and global processing deficits in nystagmus.^6^ This study investigated both local and global deficits in nystagmus associated with albinism using Gabor patches.

We employed two experimental paradigms in the current study, coherence threshold^22^ and equivalent noise,^23^ to investigate the role of both local and global limits on motion and form perception in nystagmus. The coherence threshold represents the minimum proportion of elements that follow the coherent direction and orientation (such as left or right) necessary to accurately judge global percept in the presence of random directional/orientational noise elements.^22^ Although this provides a useful index of performance, it cannot indicate whether the deficits originate at local or global levels of processing. In the equivalent noise paradigm, the local direction and orientation of individual elements are derived from a standard Gaussian distribution with prescribed mean and standard deviation.^23^^,^^24^ The external noise can be varied by changing the standard deviation of the distribution. The discrimination thresholds measured at varying levels of external noise can then be used to derive internal noise and sampling efficiency parameters that represent indices of local and global processing, respectively.^24^^,^^25^ Hence, using both paradigms can provide overall sensitivity and limitations at local and global processing levels of motion and form processing in nystagmus.

In this study, we used physically equivalent stimuli along the motion and form domains to compare performance between nystagmus and normal controls. We measured motion and form sensitivity using the coherence threshold and equivalent noise paradigms to investigate any difference in performance at local and global processing levels.

## Materials and Methods

### Participants

Thirty individuals with INS (14.84 ± 4.84 years old) and 30 age-matched controls were recruited from the outpatient department at the Himalaya Eye Hospital, Pokhara, Nepal. The protocol of the study was approved by the Nepal Health Research Council, and informed consent was obtained from all participants and/or their guardians.

The diagnosis of INS was established by a pediatric ophthalmologist (author HBA) based on documented onset in early infancy, characteristic clinical features, and comprehensive ophthalmic examination. All participants underwent visual acuity (VA) testing, refraction, ocular motility assessment, stereoacuity test, anterior and posterior segment evaluation, and optical coherence tomography of the retina and optic nerve. Participants were classified as having “idiopathic INS” only when no associated ocular or systemic condition (e.g., optic nerve hypoplasia, aniridia, retinal dystrophy, or neurological disease) was identified through these assessments.

All participants were also assessed clinically for features of periodic alternating nystagmus (PAN) by observing spontaneous changes in the fast direction under binocular viewing. Features of fusion maldevelopment nystagmus syndrome (FMNS) were also evaluated using cover and duction tests. The INS group included 20 individuals with idiopathic nystagmus, eight associated with oculocutaneous albinism, and two following congenital cataract. Clinical details are summarized in the Table.

**Table.** Clinical Details for Nystagmus Participants

				VA (logMAR)		
ID	Age (Y)	Sex	Nystagmus Diagnosis	Right Eye	Left Eye	Stereoacuity (Arcsec Frisby Test)
1	7	M	Idiopathic	0.46	0.44	150
2	17	M	Idiopathic	0.46	0.46	85
3	9	M	Idiopathic	0.76	0.66	215
4	12	F	Idiopathic	0.9	0.9	215
5	8	M	Congenital cataract	0.94	0.78	300
6	20	M	Albinism	1.24	1.24	600
7	13	F	Idiopathic	0.26	0.2	20
8	16	M	Idiopathic	0.26	0.32	75
9	27	F	Idiopathic	1.02	1	170
10	7	M	Idiopathic	0.7	0.44	170
11	10	M	Idiopathic	0.66	0.76	215
12	13	M	Idiopathic	0.86	0.86	150
13	8	F	Idiopathic	0.46	0.38	300
14	17	F	Albinism	0.92	0.94	170
15	16	F	Albinism	1	1.06	300
16	17	M	Albinism	0.86	0.72	110
17	15	F	Albinism	0.7	0.76	110
18	19	F	Idiopathic	0.14	0.14	40
19	13	F	Albinism	0.82	0.84	40
20	16	F	Albinism	0.78	0.72	170
21	11	M	Idiopathic	0.52	0.54	170
22	12	F	Idiopathic	0.5	0.52	40
23	22	M	Idiopathic	0.86	0.82	85
24	17	F	Congenital cataract	0.54	0.56	55
25	10	F	Albinism	1.12	1.12	85
26	13	M	Idiopathic	0.72	0.7	40
27	16	M	Idiopathic	1.22	0.86	55
28	23	F	Idiopathic	0.42	0.42	55
29	16	F	Idiopathic	0.44	0.46	30
30	12	M	Idiopathic	0.54	0.5	30

VA was measured monocularly using an Early Treatment Diabetic Retinopathy Study (ETDRS) chart under normal room illumination at 4 meters, with the fellow eye fogged and no time restrictions. If participants were unable to read the logMAR 1.0 line at 4 meters, the test distance was reduced to 2 meters, and scores were adjusted accordingly. All control participants demonstrated unaided or corrected VA of 0.00 logMAR and normal ocular health aside from refractive error. Stereoacuity was measured using the Frisby test.

### Stimuli

The stimuli consisted of physically equivalent translational RDKs and Glass patterns. These were based on stimuli that have been described in our previous studies.^14^^,^^26^ Briefly, the stimuli were generated using MATLAB 2022 (MathWorks, Natick, MA, USA) with Psychophysics Toolbox extensions^27^^,^^28^ on an ASUS ExpertBook laptop (ASUS, Taipei, Taiwan) with an Intel CORE i5 processor (Intel Corporation, Santa Clara, CA, USA) and a monitor refresh rate of 75 Hz. Both stimuli consisted of 240 black dots (0.167° in diameter, dot density of 10.52 dots/deg^2^) presented in a circular aperture (8° in diameter) at a display distance of 50 cm. The mean background luminance was 35 cd/m^2^, and the contrast of the dot elements was 95% Michelson contrast.

### Glass Patterns

Glass patterns were created by first randomly positioning 120 dots in the circular aperture. An identical copy of the dot pattern was then superimposed after a linear transformation at 45° or 135° with a dot displacement distance of 0.266°. This created translational Glass patterns containing 120 dipole pairs.

### Random Dot Kinematograms

In the RDKs, all 240 dots were first randomly positioned in the aperture, which then moved following a linear trajectory as described for Glass patterns for six frames (0.08 second) at dot speeds of either 5°/s or 10°/s, after which they were generated at a random location within the circular aperture and continued the same trajectory. The dots that reached the edge of the aperture were randomly generated within the stimulus area.

**Figure 1.**
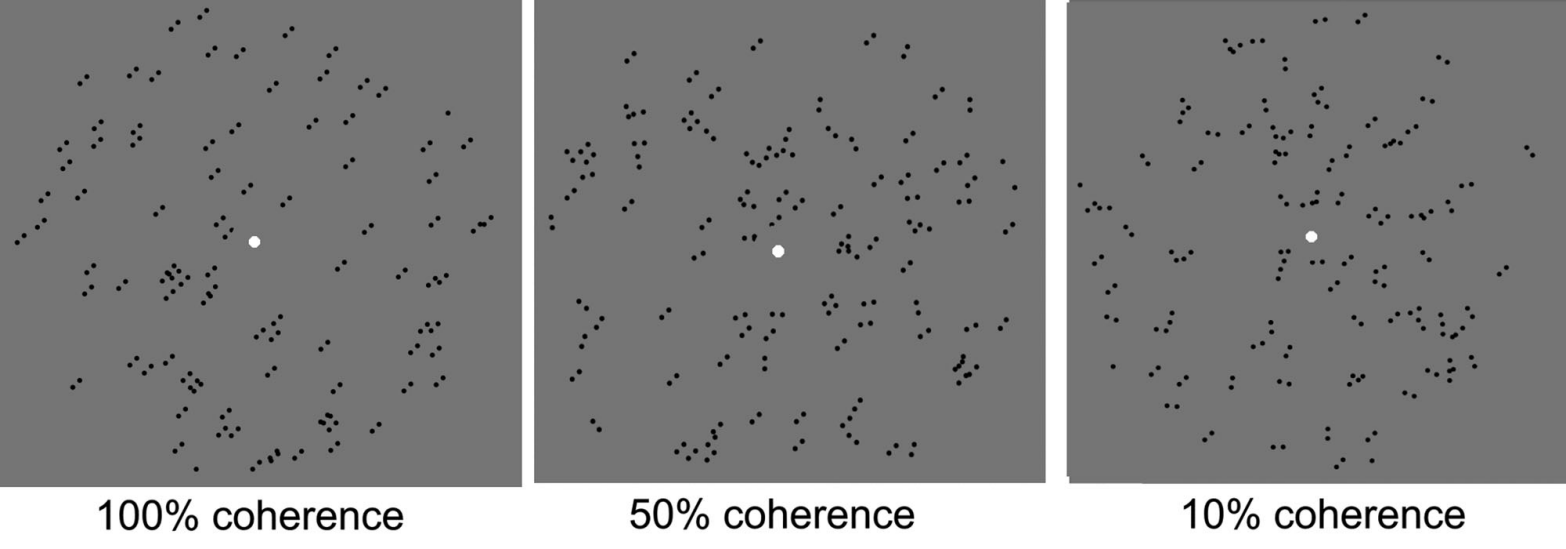
Glass patterns at different coherence levels.

Two experimental paradigms were employed: coherence threshold and equivalent noise. In the coherence threshold paradigm, signal dots (RDKs) and dipoles (Glass patterns) followed the direction of motion/orientation of 45° left or right from vertical, and the noise dots followed a random direction/orientation (360°) (Fig. 1). The global motion/orientation of the RDKs and Glass patterns were randomized, 45° left to right from vertical for each trial. For RDKs, two dot speeds, 5°/s and 10°/s, were assessed.

**Figure 2.**
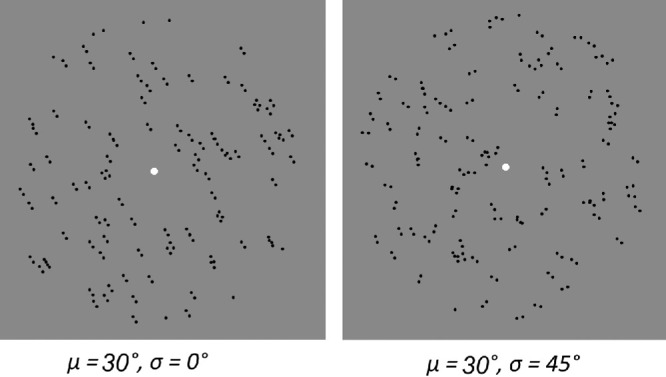
Glass patterns with individual dipole orientations generated from a standard Gaussian distribution with varying external noise (SD) and the same global orientation (mean = 30°). No noise (left); high noise (*right*).

For the external noise paradigm,^24^^,^^29^ the direction of motion/orientation of all constituent dots and dipoles was generated from a standard Gaussian distribution. The mean of the distribution represents the global direction/orientation of the pattern, and the change in standard deviation provides a measure of added external noise (Fig. 2). The change in the mean of the distribution varied the angle from the vertical reference (90°). Five external noise levels (0°, 5°, 10°, 20°, and 45°) were used at a single dot speed of 10°/s for RDKs and 0.266° dipole distance for Glass patterns.

### Procedure

The participants were seated in a dark, quiet room. At the start of the experiments for both paradigms (coherence threshold and equivalent noise), a fixation dot (0.2° diameter) appeared at the center of the screen followed by the presentation of a Glass pattern or RDK for 1.0 second. Participants were asked to look straight at the fixation dot and were not asked to adopt any head posture. Familiar cartoon characters were added to the left and right top of the screen as an indication of which direction/orientation to expect in the RDKs or Glass patterns. An 8°-diameter circular mask consisting of randomly generated pixel noise was displayed for 0.25 second after each stimulus presentation.

**Figure 3.**
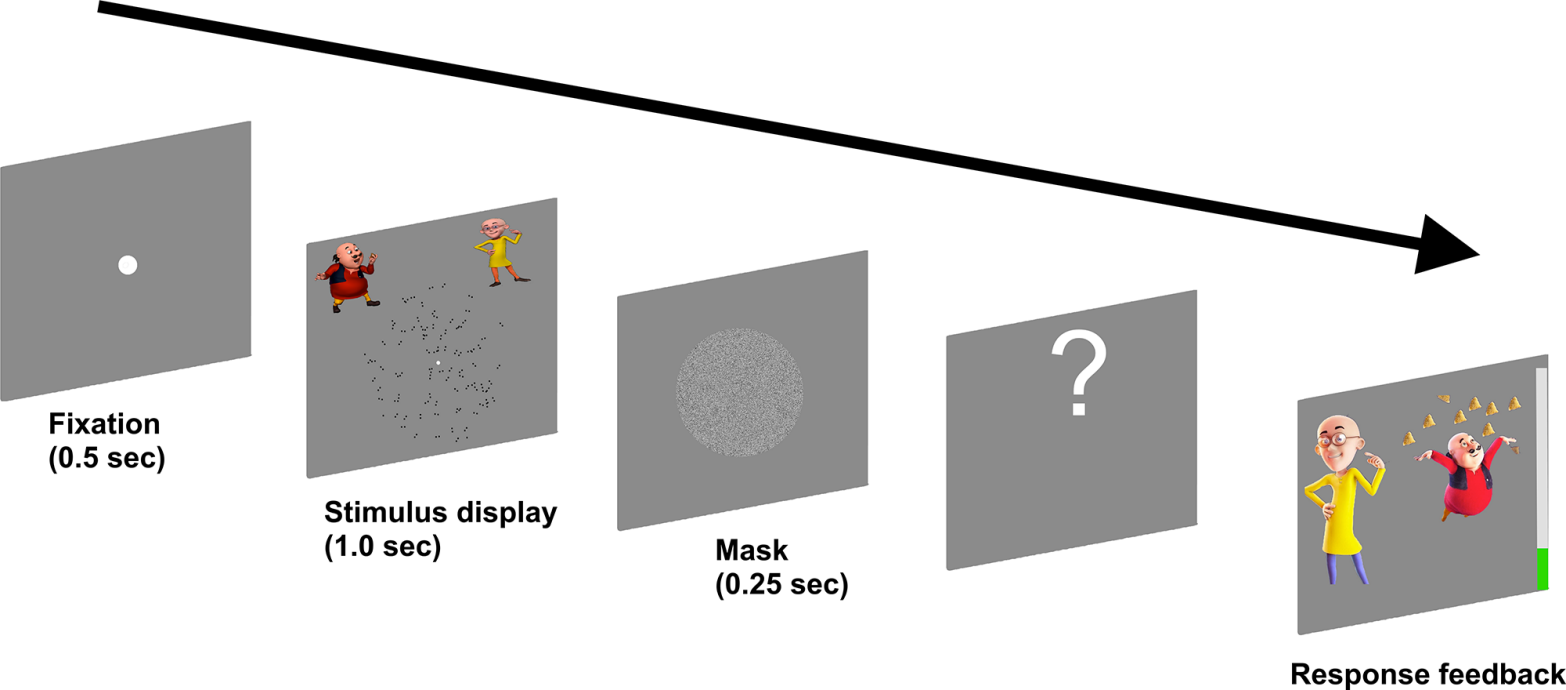
Schematic representation of a single trial.

The participant's task in both experiments was to indicate if the global direction of the motion in the RDK and the global orientation of the Glass pattern were to the left or the right of the vertical reference (90°). The participants were asked which cartoon character they thought the pattern of dots were mostly moving/orientated toward and to indicate that by pressing the respective arrow keys (left or right). Both positive and negative feedback were provided after each trial which contained happy/sad expression of the cartoon characters. An indication of the remaining trials was also provided as a progress bar at the side of the feedback screen (Fig. 3).

A 3:1 adaptive staircase was used for data collection. For the coherence threshold paradigm, each staircase started with 50% of the dots or dipoles following a coherent direction/orientation (translation to 45° to the left or right). For the equivalent noise paradigm, the staircases for external noise (five levels) were interleaved. Each staircase started with an angle of 30° from vertical. For both paradigms, the initial step size for change in the number of coherent elements (coherence threshold paradigm) and change in the angle from vertical (equivalent noise paradigm) was an octave, which was reduced to half an octave and further to a quarter of an octave after three and six reversals, respectively. All staircases terminated after 100 trials or 10 reversals, whichever occurred first, and the threshold was calculated as the mean of the last seven reversals.

At the start of each experiment, participants first completed practice sessions for both stimuli consisting of 15 trials for each staircase. Only the participants who achieved 60% of correct responses in the practice sessions were further included in the study. Three people with INS were excluded, as they could not achieve this criterion despite two runs of practice sessions.

### Modeling

The discrimination thresholds (τo) at five external noise levels (σ_ext_) from the equivalent noise paradigm were fitted to the linear amplifier model (LAM)^24^ to separate the performance in terms of internal noise (σ_eq_) and sampling efficiency (*Eff*):

(1) $$\begin{array}{c} \tau_{o}=\sqrt{\frac{\sigma_{ext}+\;\sigma_{eq}}{Eff}} \end{array}$$

The full model consisted of independent internal noise and sampling efficiency parameters for the nystagmus and control groups. Two levels of nested models were generated from the full model. In the first level, either internal noise or the sampling efficiency parameter was constrained across the two groups. In the model with the internal noise constrained, the difference in performance was represented by the change in the sampling efficiency parameter; for the model with sampling efficiency constrained, the difference was due to a change in the internal noise parameter. In the final model, both internal noise and sampling efficiency parameters were constrained; this model represents the simplest model with no difference in parameters between the nystagmus and control groups.

The best model to describe the threshold data was selected by testing the goodness of fits between the nested models hierarchically with the following equation:

(2) $$\begin{array}{c} F\left(df_{1},\;df_{2}\right)=\;\frac{\left(r_{full}^{2}-r_{constrained}^{2}\right)/df_{1}}{\left(1-r_{full}^{2}\right)/df_{2}} \end{array}$$

where *df*_1_ = *k_full_* – *k_constrained_* and *df*_2_ = *N* – *k_full_*, where *k* is the number of parameters in each model, and *N* is the number of predicted data points.

## Results

No clinical evidence of PAN or FMNS was observed in any of the participants with INS in this study. The mean VA of participants with INS (0.687 ± 0.275) was worse compared to normal controls (0.007 ± 0.056), *t*(64) = 18.75, *P* < 0.001. Similarly, the mean stereoacuity was also poorer in INS (141.66 ± 121.09 arcsec) compared to the normal controls (14.67 ± 7.98 arcsec), *t*(29) = 5.73, *P* < 0.01.

### Coherence Threshold Paradigm

**Figure 4.**
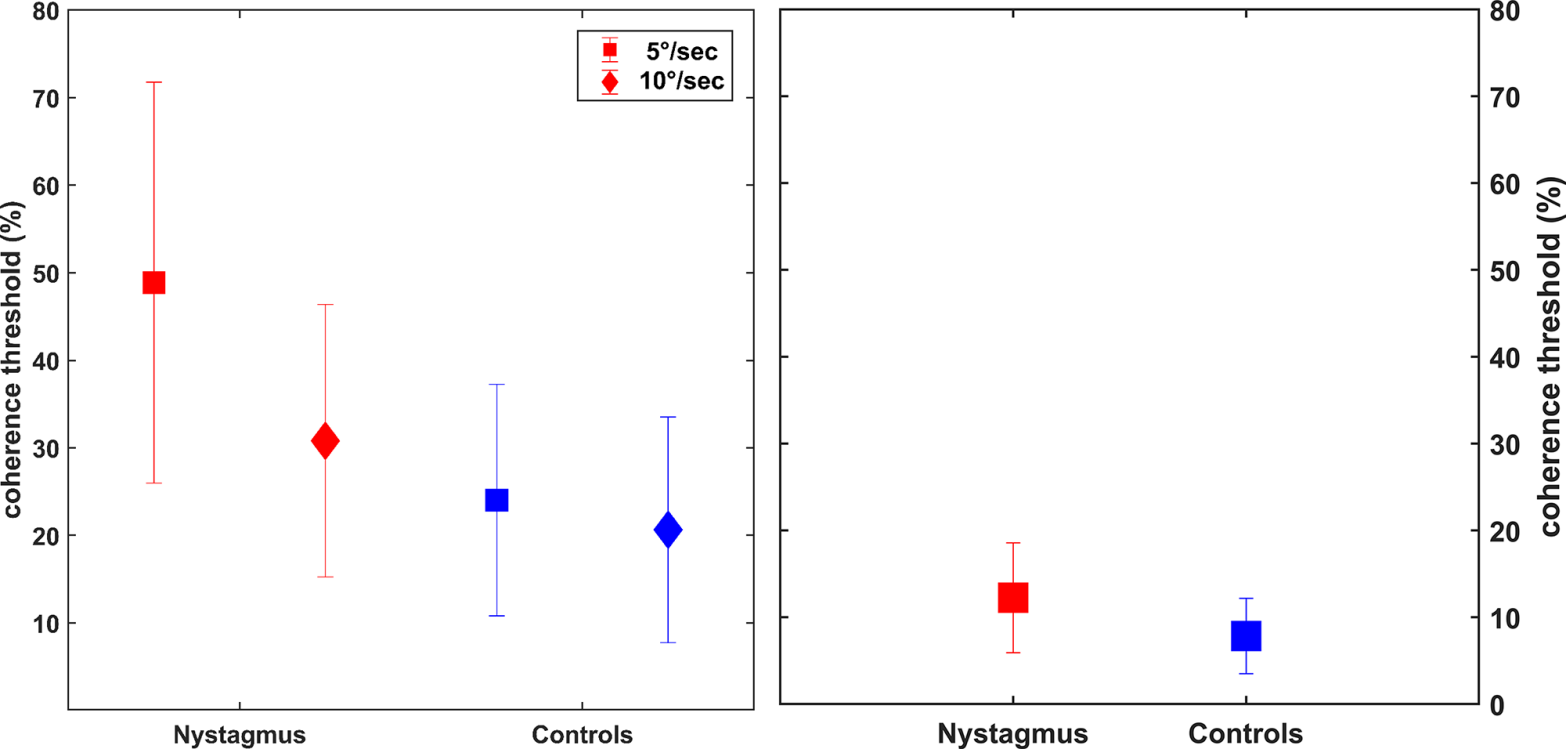
Coherence threshold for RDKs (*left*) and Glass patterns (*right*). *Error bars* represent SD.

The mean motion coherence thresholds (MCTs) for RDKs were higher for the INS group (5°/s: 50.55% ± 21.33%; 10°/s: 31.87% ± 14.69%) compared to the controls (5°/s: 24.04% ± 13.23%; 10°/s: 20.65% ± 2.89%). The difference was analyzed with two-way ANOVAs with speed (5°/s and 10°/s) and participants (nystagmus and controls) as fixed factors. The results showed that both effect of speed, *F*(1, 114) = 14.28, *P* < 0.01, and participants, *F*(1, 114) = 41.74, *P* < 0.01, were statistically significant. The interaction between the speed and participants was also significant, *F*(1, 114) = 6.86, *P* = 0.01. On pairwise comparison, the thresholds for nystagmus were higher than those of the controls (*P* < 0.01), and the threshold at 5°/s were higher than those at 10°/s (*P* < 0.01). The borderline interaction was due to similar thresholds for controls at 2 speeds (5°/s: 24.04% ± 13.22%; 10°/s: 20.65% ± 12.89%), but for nystagmus the thresholds for slower speed (50.55% ± 21.33%) were higher than for the faster speed (31.87% ± 14.88%). Similarly, the mean orientation threshold for Glass patterns was also higher for INS (12.23% ± 0.32%) compared to controls (7.84% ± 0.33%), *t*(51) = 3.14, *P* < 0.01 (Fig. 4).

To assess the extent of INS deficits at two speeds for RDKs, we calculated the index of deficit for each participant (individual INS MCT/mean control MCT) at both speeds. The mean deficit index for the slower speed of 5°/s (2.10 ± 0.89) was higher compared to the speed of 10°/s (1.54 ± 0.71), *t*(28) = 3.31, *P* < 0.001. The deficit index for the Glass patterns (1.56 ± 0.80) was similar to the faster speed.

### Equivalent Noise Paradigm

**Figure 5.**
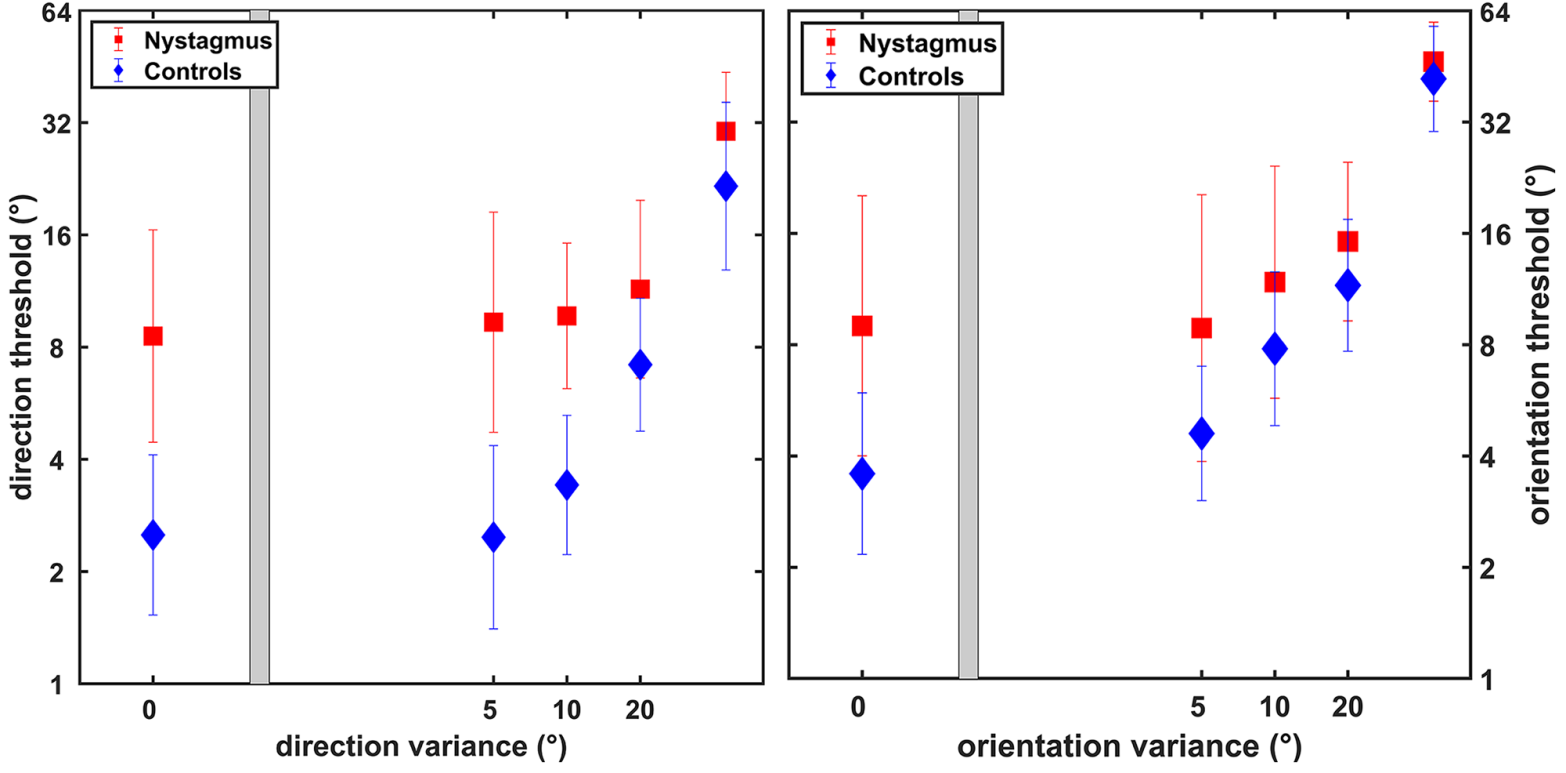
Discrimination thresholds at five external noise levels; RDKs (*left*) and Glass patterns (*right*). *Error bars* represent SD.

For both RDKs and Glass patterns, the thresholds were low for no noise conditions and increased with increasing external noise. For both stimuli, the thresholds for INS were higher compared to the control group at no noise and lower noise levels. At the highest noise level (45°), the thresholds for INS were similar to those of the control group (Fig. 5).

A mixed-method ANOVA was used to investigate the effect of noise levels (within-subject factor) and INS versus control groups (between-subject factor) on discrimination thresholds. The results showed that the effect of the within-subject factor (noise levels) was significant for RDKs, *F*(4, 53) = 51.25, *P* < 0.001, and Glass patterns, *F*(4, 44) = 124.83, *P* < 0.001, but there was no significant interaction between within subject factor (noise levels) or between subject factor (nystagmus vs. controls; *P* > 0.05). The effect of the between-subject factor (nystagmus vs. controls) was also significant for both RDKs, *F*(1, 56) = 402.09, *P* < 0.001, and Glass patterns, *F*(1, 47) = 398.26, *P* < 0.001. On pairwise comparison after Bonferroni correction, the thresholds for RDKs at lower and mid noise levels were significantly different between nystagmus and controls (*P* < 0. 01), but the difference was not statistically significant at the highest noise level (45°) (*P* > 0.01). Similarly, the thresholds for Glass patterns were higher for nystagmus at low and mid noise levels (*P* < 0.05), but the difference was not statistically significant at the highest noise level (*P* > 0.05).

**Figure 6.**
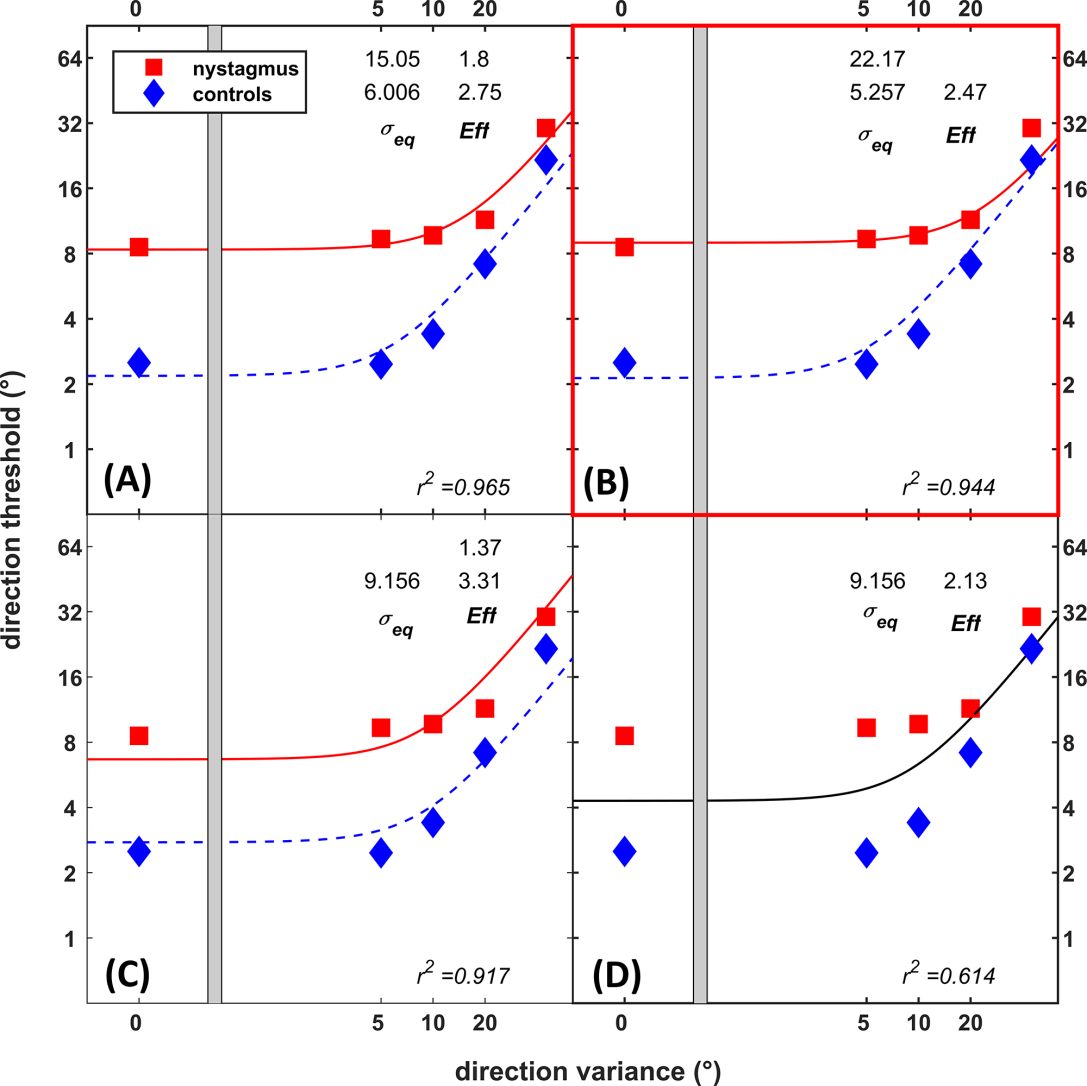
Nested models for direction discrimination thresholds (RDKs) for nystagmus and controls. The full model (**A**) was constrained to create different nested models with *Eff* constrained (**B**), σ_eq_ constrained (**C**), or both constrained (**D**). The model with *Eff* constrained was the best model.

**Figure 7.**
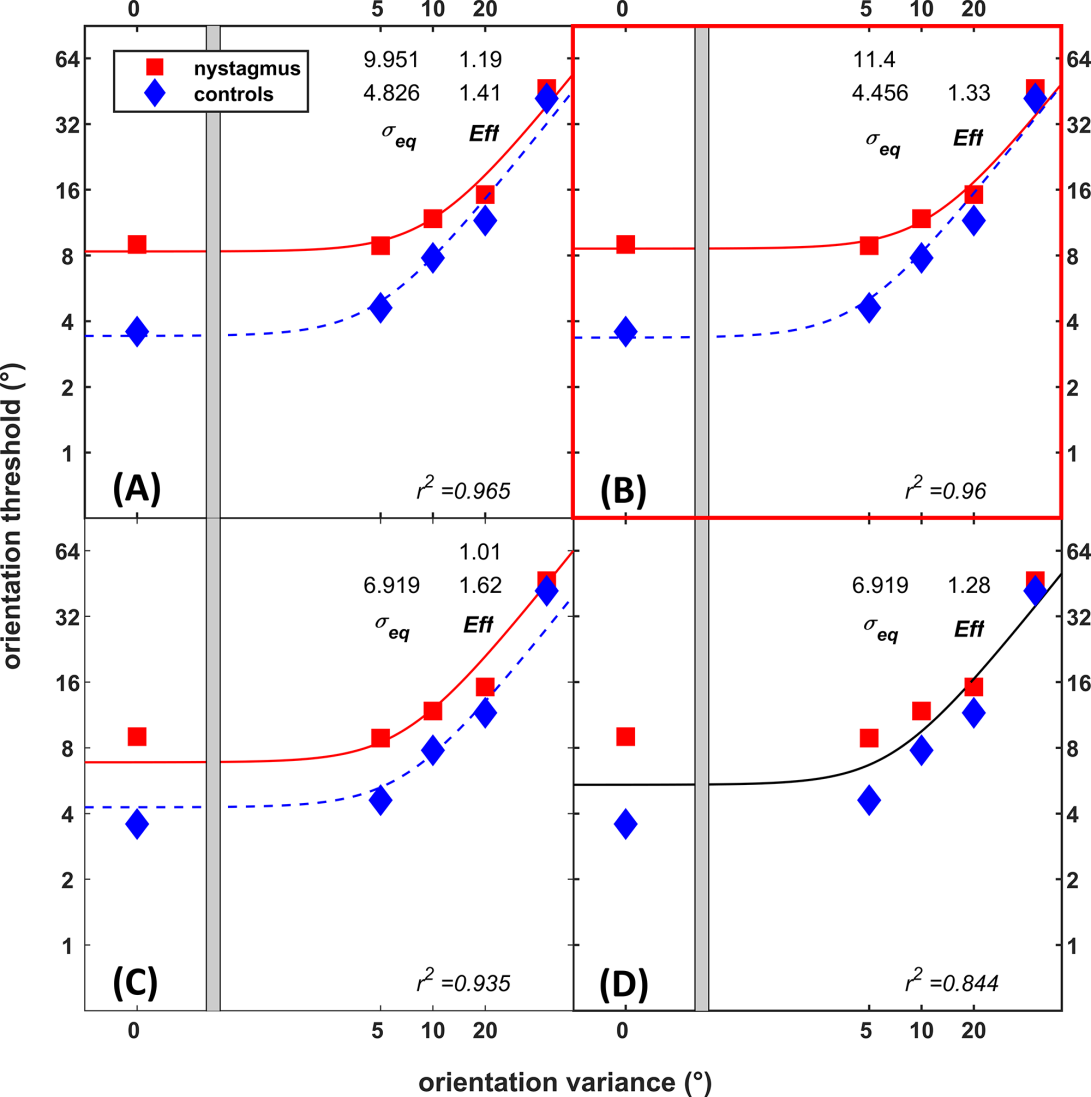
Nested models for orientation discrimination thresholds for nystagmus and controls. The full model (**A**) was constrained to create different nested models with *Eff* constrained (**B**), σ_eq_ constrained (**C**), or both constrained (**D**). The model with *Eff* constrained was the best model.

The discrimination thresholds for RDKs and Glass patterns were fitted to the LAM (Equation 1). The full model contained independent internal noise (σ_eq_) and sampling efficiency (*Eff*) parameters for INS and controls (Figs. 6, 7A). Three restricted nested models were created by constraining free parameters (σ_eq_ and *Eff*)*.* The goodness-of-fit statistics (*r*^2^) for all nested models were compared to the full model (Equation 2). For RDKs (Fig. 6), the model with sampling efficiency constrained, *r*^2^ = 0.944, *F*(1, 6) = 3.60, *P* = 0.11, was statistically similar to the full model (*r*^2^ = 0.965). The model with internal noise constrained, *r*^2^ = 0.917, *F*(1, 6) = 8.23, *P* < 0.05, showed poorer fit compared to the full model. The simplest model with both σ_eq_ and *Eff* constrained meanwhile showed poorer fit (*r*^2^ = 0.614) compared to the full model, *F*(2, 6) = 30.08, *P* < 0.001, and to both constrained models (*P* < 0.01). Hence, the model with sampling efficiency constrained and difference in performance due to change in the internal noise was chosen as the best model.

For Glass patterns (Fig. 7), models with *Eff* constrained, *r*^2^ = 0*.*960, *F*(1, 6) = 0*.*86, *P* = 0*.*39, and σ_eq_ constrained, *r*^2^ = 0*.*935, *F*(1, 6) = 5*.*14, *P* = 0*.*06, were statistically similar to the full model (*r*^2^ = 0*.*965). The simplest model (*r*^2^ = 0*.*844) showed poorer fit compared to the full model, *F*(2, 6) = 10*.*37, *P* < 0*.*05, and to both constrained models (*P* < 0*.*01). Because both models with *Eff* (*r*^2^ = 0*.*960) and σ_eq_ (*r*^2^ = 0*.*935) constrained were similar to the full model, the model with sampling efficiency constrained and difference in performance due to change in the internal noise was chosen as the best model based on the better goodness of fit measure (*r*^2^).

## Discussion

Using two experimental paradigms, we showed that INS results in reduced sensitivity to both global motion and global form. For global motion, the coherence thresholds were elevated for INS at both 5°/s and 10°/s speeds. Furthermore, employing an equivalent noise paradigm, we showed that the thresholds at no noise and lower noise are elevated in INS and the differences in performance compared to normal controls for both global motion and global form are due to higher internal noise.

Two main causes for the spectrum of deficits observed in INS have been discussed in previous studies: retinal image smearing because of abnormal eye movements and cortical changes due to early visual deprivation.^4^^,^^5^^,^^10^ Retinal blur would have a larger impact on discrimination of moving stimuli compared to the static stimuli due to the interaction of eye movement with stimuli motion. Our results for a higher coherence threshold and higher internal noise for global motion compared to the global form task are in line with previous reports of elevated motion thresholds in INS.^4^^,^^5^ If retinal blur due to eye movement is the main cause of elevated thresholds, the performance along the horizontal meridian (direction of abnormal INS eye movements) is expected to be poorer than for the vertical meridian. Higher thresholds for motion discrimination along the horizontal meridian has been reported using sinusoidal gratings,^4^ but there was no difference between the motion coherence thresholds for vertical versus horizontal motion using RDKs^5^ and Gabor patches.^6^ Similarly for crowding, some studies have reported a larger effect along the horizontal meridian, but others have reported no differences in performance.^7^ The role of eye movements has also been evaluated by assessing visual functions at the null point, where eye movements are reduced. Velocity discrimination of local motion (i.e., discriminating the velocity of two moving sinusoidal gratings) improves at the null point.^4^ However, global motion coherence deficits (evaluated using a task similar to that in the current study) are present at the null point.^5^ Additionally, adding nystagmoid waveform motion to the stimuli for normal observers increased crowding compared to without.^7^ These results suggest that the impact of abnormal eye movements observed in INS may be higher for stimuli with motion compared to static visual functions.

Our results indicate that the model with the higher internal noise for INS best explained the differences between INS and normal controls, a finding that suggest that changes in local processing mechanisms may be largely responsible for elevated thresholds. The internal noise within the global motion/form processing mechanism arises at early areas of visual processing where local information, such as the motion of individual dots and orientation of dipoles, is processed.^14^^,^^25^^,^^26^ The primary visual cortex is the proposed site of such local processing for both motion and form processing.^25^ However, degraded visual cues from the retina due to smearing in the early phases of visual development could also contribute to the upstream deficits in local processing mechanism at V1. Hence, these results support a role for early visual processing deficits, such as in the primary visual cortex, in elevated thresholds.

Although retinal smearing due to abnormal eye movements has been discussed as a cause for motion deficit in INS, this may not fully account for the pattern of deficit observed.^10^ When the deprivation period due to nystagmus is added as a factor in a model containing eye movements (nystagmus waveforms), foveation period (fixation stability), etc., this model was better at predicting VA deficits observed in nystagmus.^10^ Our modeling results indicate that, although internal noise was the main factor explaining differences in performance, as expected, the full model, which included sampling efficiency, had relatively better goodness of fit measures. Another study using the equivalent noise paradigm but employing Gabor patches reported that changes in both internal noise and sampling efficiency contributed to deficits in motion perception in INS associated with albinism.^6^ The sampling efficiency parameter is related to the global processing stage where local inputs from V1 are combined in the areas with larger receptive fields, such as MT for motion, and V4 from the global form.^25^

Another finding that supports wider impact on visual processing in INS is the presence of deficits for both global motion and global form. These two visual functions are mostly processed along independent pathways along dorsal and ventral streams.^18^ The finding that both processing mechanisms are affected in INS suggests a general impact of INS on visual function, to which early visual deprivation may be a contributor, similar to another developmental disorder, amblyopia. We have previously reported^14^^,^^30^ that both global motion and form are affected in amblyopia using stimuli similar to those in the current study. Interestingly, we found that global form is affected more in amblyopia compared to motion and these deficits were related to the difference in sampling efficiency parameter and not the internal noise as in the current study. Our results suggest that both motion and form deficits in INS are due to changes in the early processing mechanism within the visual system up to the primary visual cortex where local motion and form cues are processed. This could be due to the effects of early visual deprivation from abnormal eye movements in the developmental phase, similar to what occurs in amblyopia.

### Effect of Speed on INS Motion Perception

INS resulted in elevated motion coherence thresholds for both speeds compared to normal controls. The mean coherence threshold in the INS group was higher for the slower speed, and the deficit index was also larger for the slower speed (5°/s) compared to the faster speed (10°/s). A previous study using RDKs also reported higher coherence thresholds for INS compared to normal controls at a dot speed of 10°/s.^5^

Elevated motion discrimination thresholds have been reported to be adaptive mechanisms in INS. INS results in constant retinal image motion; however, patients with INS rarely report oscillopsia. The reduced sensitivity to motion helps to dampen the effect of constant retinal motion, thereby maintaining a stable visual world.^31^^,^^32^ The speed at which INS patients experience perceptual stability is reported to be at retinal slip of <4°/s.^4^^,^^33^^,^^34^ Another study investigating the effect of object motion on perceptual stability reported that, although normal controls experienced oscillopsia, people with INS had stable visual perception at object speeds up to 8°/s.^35^ The RDK speeds used in the current study (5°/s and 10°/s) are close to these limits, which could have resulted in reduced sensitivity at both speeds and larger effect on the slower speed.

INS results in lower sensitivity to both global motion and global form perception. For motion perception, slower speeds are affected more than faster speeds. These deficits in both motion and form domains are a result of higher internal noise at the local processing stage in early areas of the visual pathway such as V1. In the future, it would be important to examine the influence of nystagmus characteristics (e.g., foveation stability, intensity, slow phase velocity in the deficits in both domains), which were not captured in the present study.

## References

[1] Abadi RV, Dickinson CM. Waveform characteristics in congenital nystagmus. *Doc Ophthalmol*. 1986; 64: 153–167. 3608756 10.1007/BF00159990

[2] Papageorgiou E, McLean RJ, Gottlob I. Nystagmus in childhood. *Pediatr Neonatol*. 2014; 55: 341–351. 25086850 10.1016/j.pedneo.2014.02.007

[3] Fu VLN, Bilonick RA, Felius J, Hertle RW, Birch EE. Visual acuity development of children with infantile nystagmus syndrome. *Invest Ophthalmol Vis Sci*. 2011; 52: 1404–1411. 21071734 10.1167/iovs.09-4686 PMC3101699

[4] Dai B, Cham KM, Abel LA. Velocity discrimination in infantile nystagmus syndrome. *Invest Ophthalmol Vis Sci*. 2021; 62: 35. 10.1167/iovs.62.10.35 PMC8411855 34459850

[5] Dai B, Cham KM, Abel LA. Perception of coherent motion in infantile nystagmus syndrome. *Invest Ophthalmol Vis Sci*. 2022; 63: 31. 10.1167/iovs.63.1.31 PMC8802013 35072688

[6] Neveu MM, Jeffery G, Moore AT, Dakin SC. Deficits in local and global motion perception arising from abnormal eye movements. *J Vis*. 2009; 9: 1–15. 19757918 10.1167/9.4.9

[7] Tailor VK, Theodorou M, Dahlmann-Noor AH, Dekker TM, Greenwood JA. Eye movements elevate crowding in idiopathic infantile nystagmus syndrome. *J Vis*. 2021; 21: 1–23. 10.1167/jov.21.13.9 PMC8709927 34935877

[8] Chung ST, Bedell HE. Effect of retinal image motion on visual acuity and contour interaction in congenital nystagmus. *Vision Res*. 1995; 35: 3071–3082. 8533343 10.1016/0042-6989(95)00090-m

[9] Almagren B, Dunn MJ. Measurement of visual function in infantile nystagmus: a systematic review. *Br J Ophthalmol*. 2024; 108: 1038–1043. 38164583 10.1136/bjo-2023-324254

[10] Felius J, Muhanna ZA. Visual deprivation and foveation characteristics both underlie visual acuity deficits in idiopathic infantile nystagmus. *Invest Ophthalmol Vis Sci*. 2013; 54: 3520–3525. 23687170 10.1167/iovs.13-11992

[11] Harris C, Berry D. A developmental model of infantile nystagmus. *Semin Ophthalmol*. 2006; 21: 63–69. 16702071 10.1080/08820530600613746

[12] Dunn MJ, Margrain TH, Woodhouse JM, Ennis FA, Harris CM, Erichsen JT. Grating visual acuity in infantile nystagmus in the absence of image motion. *Invest Ophthalmol Vis Sci*. 2014; 55: 2682–2686. 24651552 10.1167/iovs.13-13455

[13] von Noorden GK . Amblyopia: a multidisciplinary approach. Proctor lecture. *Invest Ophthalmol Vis Sci*. 1985; 26: 1704–1716. 3934105

[14] Joshi MR, Simmers AJ, Jeon ST. Concurrent investigation of global motion and form processing in amblyopia: an equivalent noise approach. *Invest Ophthalmol Vis Sci*. 2016; 57: 5015–5022. 27654428 10.1167/iovs.15-18609

[15] Hamm LM, Black J, Dai S, Thompson B. Global processing in amblyopia: a review. *Front Psychol*. 2014; 5: 583. 24987383 10.3389/fpsyg.2014.00583 PMC4060804

[16] Braddick O, Atkinson J, Wattam-Bell J. Normal and anomalous development of visual motion processing: motion coherence and ‘dorsal-stream vulnerability’. *Neuropsychologia*. 2003; 41: 1769–1784. 14527540 10.1016/s0028-3932(03)00178-7

[17] Braddick OJ, O'Brien JM, Wattam-Bell J, Atkinson J, Hartley T, Turner R. Brain areas sensitive to coherent visual motion. *Perception*. 2001; 30: 61–72. 11257978 10.1068/p3048

[18] Milner AD, Goodale MA. Two visual systems re-viewed. *Neuropsychologia*. 2008; 46: 774–785. 18037456 10.1016/j.neuropsychologia.2007.10.005

[19] Dakin SC . The detection of structure in Glass patterns: psychophysics and computational models. *Vision Res*. 1997; 37: 2227–2246. 9578905 10.1016/s0042-6989(97)00038-2

[20] Morrone MC, Burr DC, Vaina LM. Two stages of visual processing for radial and circular motion. *Nature*. 1995; 376: 507–509. 7637781 10.1038/376507a0

[21] Wilson HR, Wilkinson F. Detection of global structure in Glass patterns: implications for form vision. *Vision Res*. 1998; 38: 2933–2947. 9797989 10.1016/s0042-6989(98)00109-6

[22] Newsome WT, Pare EB. A selective impairment of motion perception following lesions of the middle temporal visual area (MT). *J Neurosci*. 1988; 8: 2201–2211. 3385495 10.1523/JNEUROSCI.08-06-02201.1988 PMC6569328

[23] Watamaniuk SN, Sekuler R. Temporal and spatial integration in dynamic random-dot stimuli. *Vision Res*. 1992; 32: 2341–2347. 1288010 10.1016/0042-6989(92)90097-3

[24] Pelli DG, Farell B. Why use noise? *J Opt Soc Am A Opt Image Sci Vis*. 1999; 16: 647–653. 10069051 10.1364/josaa.16.000647

[25] Dakin SC, Mareschal I, Bex PJ. Local and global limitations on direction integration assessed using equivalent noise analysis. *Vision Res*. 2005; 45: 3027–3049. 16171844 10.1016/j.visres.2005.07.037

[26] Joshi MR, Simmers AJ, Jeon ST. The interaction of global motion and global form processing on the perception of implied motion: an equivalent noise approach. *Vision Res*. 2021; 186: 34–40. 34030023 10.1016/j.visres.2021.04.006

[27] Brainard DH . The Psychophysics Toolbox. *Spatial Vision*. 1997; 10: 433–436. 9176952

[28] Kleiner M, Brainard D, Pelli D. What's new in Psychtoolbox-3? *Perception*. 2007; 36: 1–16.

[29] Barlow HB . Noise and the visual threshold. *Nature*. 1957; 180: 1405. 10.1038/1801403a0 13493537

[30] Joshi MR, Simmers AJ, Jeon ST. Implied motion from form shows motion aids the perception of global form in amblyopia. *Invest Ophthalmol Vis Sci*. 2020; 61: 58. 10.1167/iovs.61.5.58 PMC7405683 32460320

[31] Shallo-Hoffmann JA, Bronstein AM, Acheson J, Morland AB, Gresty MA. Vertical and horizontal motion perception in congenital nystagmus. *Neuro-Ophthalmology*. 1998; 19: 171–183.

[32] Leigh RJ, Dell'Osso LF, Yaniglos SS, Thurston SE. Oscillopsia, retinal image stabilization and congenital nystagmus. *Invest Ophthalmol Vis Sci*. 1988; 29: 279–282. 3338885

[33] Abel LA, Williams IM, Levi L. Intermittent oscillopsia in a case of congenital nystagmus. Dependence upon waveform. *Invest Ophthalmol Vis Sci*. 1991; 32: 3104–3108. 1938285

[34] Dell'osso LF, Leigh RJ. Ocular motor stability of foveation periods. *Neuro-Ophthalmology*. 1992; 12: 303–326.

[35] Bedell HE . Perception of a clear and stable visual world with congenital nystagmus. *Optom Vis Sci*. 2000; 77: 573–581. 11138830 10.1097/00006324-200011000-00006

